# Glycemic meritocracy, sport and care: Between lay expertise and medical recognition

**DOI:** 10.1371/journal.pone.0353885

**Published:** 2026-07-14

**Authors:** Jean-Charles Vauthier, Bernard Kabuth

**Affiliations:** 1 Faculty of Medicine, Maieutics and Health Professions, Université de Lorraine, Vandoeuvre Lès Nancy, France; 2 Laboratoire INTERPSY EA 4432, Université de Lorraine, Nancy, France; 3 Centre psychothérapeutique de Laxou, Laxou, France; Japanese Academy of Health and Practice, JAPAN

## Abstract

**Objective:**

To explore the physician–patient relationship among individuals with type 1 diabetes engaged in extreme endurance sports, highlighting dynamics of recognition, autonomy, and negotiation in care.

**Methods:**

Qualitative study based on thirteen semi-structured interviews with French-speaking runners living with type 1 diabetes. Analysis followed a constructivist grounded theory approach, with inductive coding and triangulation.

**Results:**

The findings reveal a complex relational dynamic structured by gaps in medical expertise, the emergence of experiential autonomy, evolving forms of collaboration, and persistent structural constraints. These dynamics reflect a tension between standardized biomedical knowledge and situated, experience-based practices. Recognition of patient expertise appears conditional, often depending on the ability to maintain acceptable glycemic outcomes.

**Conclusion:**

This study identifies an implicit model, termed glycemic meritocracy, in which patient autonomy and legitimacy are shaped by conformity to biomedical norms. These findings highlight the need to move beyond performance-based evaluations of care and to more fully integrate experiential knowledge into clinical practice, particularly in complex or atypical contexts.

## Introduction

The relationship between individuals living with chronic illness and healthcare professionals is a central issue in contemporary medicine. In the case of type 1 diabetes, this relationship is particularly solicited: daily management of the condition requires fine coordination between treatment, nutrition, physical activity, and medical technologies. When this pathologic condition intersects with extreme endurance sports such as ultramarathons or trail running, adjustments become more complex, and interactions with caregivers are marked by tensions, misunderstandings, negotiations, and co-constructions.

Scientific literature on diabetes and physical exercise remains largely dominated by biomedical approaches, focused on insulin adjustment protocols and hypoglycemia risks [1,2]. Subjective, relational, and identity dimensions of this experience are still underexplored, especially in atypical contexts where patients develop innovative strategies, sometimes outside official recommendations [3]. Previous qualitative research has explored certain aspects of living with type 1 diabetes in ultra-endurance sports, particularly in relation to identity, self-management, and psychosocial adaptation [4].

This relational complexity is rooted in ethical traditions that question the foundations of care. Carl Rogers, pioneer of client-centered therapy, emphasized empathic listening and recognition of subjective experience [5]. Paul Ricoeur, in his reflection on narrative identity, invites us to consider the patient as a subject in search of meaning, whose illness narrative contributes to self-reconfiguration [6]. Joan Tronto, through her ethics of care, highlights care as a relational practice shaped by responsibility, justice, and recognition [7]. Emmanuel Levinas reminds us that the encounter with the ill other entails infinite responsibility, grounded in shared vulnerability [8]. These perspectives converge toward a redefinition of the therapeutic relationship as a co-constructed space, where medical knowledge cannot be dissociated from lived experience.

In the context of type 1 diabetes and ultra-endurance, this approach becomes particularly relevant: adjustments are not merely biological calculations but ongoing negotiations between medical norms, continuous bio-tracking [9,10] and bodily subjectivity.

However, despite increasing attention to patient-centered care and experiential knowledge, little is known about how these dynamics concretely shape the physician–patient relationship in extreme and atypical contexts such as ultra-endurance sports. In particular, the ways in which experiential knowledge is constructed, negotiated, and recognized within clinical interactions remain insufficiently explored.

The aim of this study is to explore the physician–patient relationship among individuals living with type 1 diabetes engaged in extreme endurance sports, with a specific focus on the dynamics of recognition, autonomy, and negotiation in care.

More specifically, this study addresses the following research question: how do individuals with type 1 diabetes involved in ultra-endurance sports experience and negotiate their relationship with healthcare professionals, and how is their experiential knowledge acknowledged within this relationship?

## Materials and methods

### Study design

This study adopted a qualitative approach informed by constructivist grounded theory [11], which emphasizes the co-construction of meaning between researchers and participants and the iterative development of conceptual categories grounded in empirical data [12].

### Researcher positioning

The principal investigator is a general practitioner, ultra-endurance runner, and lives with type 1 diabetes. This “insider” position facilitated access to the field and rapport with participants, while also requiring sustained reflexive attention to potential biases. A reflexive research journal was maintained throughout the study to document assumptions, analytical decisions, and the evolution of interpretations [13]. Regular discussions within the research team supported critical examination of emerging categories.

### Participants and recruitment

Participants were recruited via peer support groups, social media, and word-of-mouth. We used purposive sampling with maximum variation (age of diabetes, and type of sport) to capture a range of experiences. Inclusion criteria: age >18 years, diagnosis of type 1 diabetes, completion of at least one marathon or ultra-endurance race (road or trail) within the past five years, French-speaking, and able to provide informed consent.

The final sample included 13 participants (11 men, 2 women), aged 26–66, with an average diabetes duration of 18.7 years. Recruitment was iterative and informed by emerging analysis consistent with a constructivist grounded theory approach.

### Data collection

Semi-structured interviews were conducted via videoconference between July and December 2024. Interviews were audio-recorded with participants’ consent and transcribed verbatim. An interview guide explored participants’ sporting trajectories, diabetes management practices, interactions with healthcare professionals, and perceived challenges and adaptations. Field notes and contextual observations were documented alongside transcripts. Audio files were deleted after transcription, in accordance with ethical standards.

### Data analysis

Data analysis followed the iterative steps of constructivist grounded theory, including line-by-line initial coding, focused coding, constant comparison, and category development. Analytical memos were written throughout the process to support conceptual development.

Coding was conducted collaboratively within the research team, with regular discussions to compare interpretations and refine emerging categories. NVivo® software (v1.7.2) was used to support data organization and coding. Sampling and analysis proceeded concurrently until interpretative sufficiency was reached, defined as the point at which additional data no longer provided substantively new insights into emerging categories [14,15].

### Ethical considerations

The study received approval from the Sud-Est VI ethics committee (reference: 24.00907.000253) on April 5, 2024. All participants provided written informed consent after receiving detailed information about the study’s objectives and procedures.

## Results

### Study overview

Thirteen interviews were conducted between July and December 2024 with runners living with type 1 diabetes and engaged in endurance or ultra-endurance sports. Participants, identified by codes S1 to S13, represented a diversity of ages, diabetes duration, and athletic backgrounds. The majority were men (n = 11), with an average age of 42.8 years (standard deviation: 10.8) and an average diabetes duration of 18.7 years (standard deviation: 9.8). All had completed at least one marathon or ultra-endurance race within the past five years. The predominance of male was not specifically sought and is addressed in the limitations section.

Table 1 presents the main characteristics of the participants.

**Table 1:** Characteristics of study participants.

ID	Sex	Age	Age at diagnosis	Duration of T1D	Treatment	CGM	HbA1c	Sports practiced	Max distance
**S1**	M	36	21	15	Pump (Omnipod)	Dexcom	7.8	Ultra-trail, ski, tennis-marathon	200 km
**S2**	M	26	15	11	MDI (pens)	Libre 2	N/A	Trail, triathlon, rugby-marathon	80 km
**S3**	M	53	38	15	Pump (Omnipod Dash)	Dexcom	N/A	Ultra-trail-marathon	108 km
**S4**	M	48	25	23	Pump (Omnipod, Diabeloop planned)	Dexcom G6	7.4	Trail, marathon	59 km
**S5**	F	49	23	27	Pump	Dexcom	5.8	Trail, ultra cycling, Marathon, triathlon	42 km
**S6**	M	41	19	22	Pump	Dexcom	N/A	Trail-marathon	50 km
**S7**	M	66	40	26	MDI (pens)	Libre	6.7	Trail-marathon	50 km
**S8**	M	37	13	24	Pump	Dexcom	N/A	Trail-marathon	60 km
**S9**	M	46	42	4	MDI (pens)	Libre	N/A	Trail-marathon	42 km
**S10**	M	38	10	28	Pump	Dexcom	6.4-6.5	Trail-marathon	100 km
**S11**	M	44	19	25	Pump	Dexcom	6-6.2	Trail-marathon	80 km
**S12**	M	26	23	3	MDI (pens)	Libre	N/A	Trail-marathon	42 km
**S13**	F	46	16	30	Pump	Dexcom	N/A	Trail-marathon	50 km

This table summarizes demographic and clinical characteristics of participants, including age, diabetes duration, treatment type, technology use, and athletic background. HbA1c values were not systematically collected; they are reported only when spontaneously mentioned by participants during interviews, in accordance with the protocol approved by the ethics committee.

### Thematics overview

The narratives of runners with type 1 diabetes engaged in extreme endurance sports reveal a complex relational dynamic with healthcare professionals. Beyond the biomedical focus on technical adjustments and risk prevention, participants describe an ongoing process of negotiation between medical knowledge, lived experience, and context-dependent adaptive strategies.

Four interrelated thematic axes emerged from the analysis: (1) Lack of specific medical expertise and restrictive advice; (2) Experiential autonomy and empirical learning; (3) Evolving forms of collaboration and mutual recognition; and (4) Persistent structural and institutional challenges.

These themes should not be understood as independent categories but as interconnected dimensions of a broader relational process. Together they describe a dynamic trajectory from perceived gaps in medical expertise to the development of experiential autonomy, which may, under certain conditions, evolve into more collaborative relationships. However, this evolution remains shaped and sometimes constrained by structural and institutional barriers.

Rather than opposing medical and lay knowledge, these findings highlight the conditions under which experiential knowledge is constructed, negotiated, and recognized within clinical interactions, sometimes positioning patients as primary experts of their own condition.

### Thematic development

1. Gaps in Expertise and Restrictive Attitudes Among Healthcare Professionals

The narratives consistently point to a perceived gap between the standardized knowledge held by healthcare professionals and the situated, experience-based expertise required to manage type 1 diabetes in ultra-endurance context. This misalignment is not only technical but also relational, shaping how patients perceive the legitimacy and usefulness of medical input daily practice.

#### Perceived lack of expertise.

Several participants described a sense of disconnect between their needs and the competencies of their caregivers, particularly regarding the physiological and practical complexities of prolonged physical effort. Sporting experience appears to be only marginally integrated into traditional medical knowledge, especially when healthcare professionals lack direct familiarity with these practices. As S1 notes:

*“Diabetologists don’t have that kind of information because they lack the feedback. It’s a personal construction.”*

This perceived gap is sometimes reinforced by the absence of lived experience among clinicians, which participants interpret as a limitation in understanding the subtleties of diabetes management in extreme conditions:

*“If the doctor isn’t diabetic, he doesn’t grasp all the subtleties, even with the best intentions.”* (S13)

In some cases, this limitation is explicitly acknowledged by healthcare professionals themselves:

*“There’s no one currently able to support him in our area.”* (S4, quoting his diabetologist)

Together, these accounts suggest that medical expertise is not rejected per se but is perceived as incomplete or insufficiently adapted to atypical contexts, leading patients to relativize its authority.

#### Advice perceived as generic and under-specified.

Participants frequently described the medical advice they received as overly generic and insufficiently tailored to the demands of endurance practice. Recommendations such as:

*“Be careful, take what you need if necessary” or “check regularly.”* (S8)

or consultations reduced to reaffirmation without specific guidance:

*“Keep doing what you’re doing!”* (S10),

were perceived less as meaningful clinical support than as minimal validation of patient-led practice.

Even when more concrete instructions were provided, they were often treated as provisional frameworks to be adjusted:

*“Cut insulin in half before the race,”* says S12,

before adding:

*“I adjust that my own way, based on how I feel, my experience, and my constraints.”*

This suggests that standardized recommendations function less as prescriptive rules than as starting points for individualized adaptation, reinforcing the central role of experiential calibration.

#### Initial attitudes of caution or restriction.

Several runners reported encountering cautious, and sometimes restrictive attitudes toward their sporting practices. These attitudes reflect a risk-averse orientation grounded in biomedical concerns, particularly hypoglycemia, but are experienced by patients as limiting or misaligned with their capabilities:

*“No, no! That’s dangerous! You’ll get hypos!”* (S13)

Such reactions may produce moral or affective tensions, described as “*fearful”* and “*very guilt-inducing”* (S5), and can lead patients to distance themselves from medical advice or even deliberately transgress it:

*“We weren’t supposed to run in the Bois de Vincennes, but I went anyway.”* (S6).

These findings suggest that restrictive guidance may paradoxically weaken the therapeutic relationship, by undermining trust or pushing patients toward autonomous decision-making outside clinical supervision.

#### Tensions around non-conventional practices.

The gap between medical norms and lived practices becomes particularly visible when patients adopt non-conventional strategies, such as the ketogenic diet or alternative insulin management approaches. These practices often provoke strong reactions from healthcare professionals, ranging from skepticism to outright rejection:

*“That’s impossible, you’re going to die!”.*

*”Well, that can’t be true, I’ve been doing it for 8 months, and I’m standing right in front of you!”* (S9).

For participants, such responses illustrate a perceived rigidity in medical frameworks and a limited capacity to integrate emerging or experiential forms of knowledge. However, these tensions are not uniform. Some participants describe more flexible attitudes, with clinicians willing to tolerate or even support alternative practices once their effectiveness is demonstrated:

*“He accepted that way of doing things”* (S5).

This variability highlights that the gap in expertise is not fixed but may evolve depending on individual clinicians and the outcomes achieved by patients.

Taken together, these findings indicate that the perceived lack of specific medical expertise and the predominance of cautious or normative approaches contribute to a partial disjunction between clinical recommendations and lived practice. This disjunction appears to play a key role in initiating processes of experiential learning and autonomy, which are explored in the following theme.

2. Experiential Autonomy and Empirical Learning

In response to the perceived limitations of standardized medical expertise, participants describe the development of a form of experiential autonomy grounded in empirical observation, iterative experimentation, and collective knowledge exchange. This autonomy does not emerge as an ideological rejection of medical authority, but rather as a pragmatic adaptation to complex and insufficiently codified situations.

#### Self-experimentation as situated knowledge production.

Deprived of tailored or context-specific guidance, participants engage in systematic processes of self-experimentation, transforming their own bodies and practices into sites of knowledge production. This involves repeated trial-and-error, close monitoring of physiological responses, and the progressive refinement of individualized strategies

As S1 explains:

*“I learned to record in a table… if I eat a cereal bar, a fruit paste or something else at time T, what’s the impact on my blood sugar after 30 minutes, 1 hour, 2 hours… and the same for my insulin”.*

Similarly, S9, describes a largely self-directed learning process following diagnosis:

*“I tested with food, injected myself, watched the numbers go up, went out to exercise, came back. Okay, that works, that doesn’t… I really learned my diabetes!”*

These practices suggest a shift from passive adherence to medical prescriptions toward active, experience-based calibration. Knowledge is not simply received but produced through situated engagement, with each individual constructing a personalized “operational model” of their condition.

#### The central role of patient communities.

Beyond individual experimentation, participants emphasize the importance of peer communities—particularly online—in accessing practical knowledge and validating experiential insights. These communities function as informal knowledge networks, where strategies are shared, compared, and collectively refined. S8 notes:

*“I learned much more from the group”.*

This highlights a redistribution of epistemic authority, in which peers may be perceived as more relevant sources of information than healthcare professionals in specific contexts. These spaces also provide emotional support and social recognition, helping to legitimize practices that may diverge from medical norms and reducing the sense of the isolation often associated with chronic illness.

At the same time, reliance on peer-generated knowledge introduces variability in practices and levels of expertise, suggesting that such communities operate as both resources and sites of negotiation regarding what constitutes *“valid”* or *“effective”* strategies.

#### Autonomy as a situated and relational process.

The autonomy described by participants is neither absolute nor detached from the healthcare system. Rather, it emerges as a situated process, shaped by ongoing interactions with medical advice, technological tools, and collective experiences, as illustrated by participants who simultaneously rely on clinical input while developing their own adaptive strategies.

In this configuration, autonomy is less about independence than about the capacity to navigate, interpret, and selectively integrate multiple sources of information. As suggested by participants’ accounts of adapting medical recommendations to their own experience, medical guidance is not rejected but reinterpreted considering embodied experience and contextual constraints.

However, this process is not uniform across participants. While many describe a strong capacity for self-management, others express uncertainty or dependence on external validation, pointing to variations in resources, confidence, and access to information.

Taken together, these findings suggest that experiential autonomy constitutes a central mechanism through which patients respond to the limitations of standardized care. This dynamic lays the groundwork for potential transformations in the therapeutic relationship, which may evolve toward more collaborative forms as explored in the following theme.

3. Toward Collaboration and Conditional Recognition

While experiential autonomy emerges as a response to the limitations of standardized care, participants also describe situations in which their relationship with healthcare professionals evolves toward more collaborative forms. However, this shift is neither automatic nor unconditional. Rather, it appears to depend on the recognition of patient competence, often mediated by observable biomedical outcomes.

#### Flexibility conditioned by outcomes.

Several participants report that healthcare professionals adopt a more flexible and supportive attitude once the patient demonstrates effective self-management, particularly though stable or satisfactory glycemic indicators. As S2 explains:

*“She gives me a lot of freedom as long as things are going well and my test results are good.”*

Similarly, S6 describes a progressive change in his physician’s stance, initially hesitant about his participation in a marathon, but ultimately supportive after repeated successful experiences. These accounts suggest that trust is not granted a priori but is progressively earned through demonstrable results. In this context, biomedical indicators function not only as clinical tools but also as markers of credibility, shaping the degree of autonomy that patients are allowed to exercise.

#### Recognition of experiential expertise.

In some cases, this conditional trust leads to explicit recognition of the patient’s expertise. Certain participants describe interactions in which healthcare professionals acknowledge the limits of their own knowledge in specific contexts and recognize the value of patient experience. As S3 recalls:

*“You’ll become a specialist in diabetes, and there are topics you’ll know better than I do”.*

Others report more symmetrical relationships, where exchanges are perceived as balanced contributions rather than unidirectional guidance

*“I contribute as much as he does.”* (S1)

In these situations, the traditional hierarchy of knowledge is partially reconfigured, with experiential knowledge gaining legitimacy within the clinical interaction.

#### Co-construction of management strategies.

Some participants describe forms of genuine collaboration, characterized by shared decision-making and joint problem-solving. As S3 notes:

*“We looked for answers together”.*

Healthcare professionals may also act as facilitators, directing patients toward peer networks or acknowledging external sources of expertise:

*“Reservoirs of experiences”* (S1).

These practices illustrate a shift toward a more dialogical model of care, in which knowledge circulates across multiple sources and is collectively shaped.

However, this collaborative dynamic does not extend uniformly to all patients. It tends to emerge in specific contexts, particularly when patients can demonstrate consistent and *“acceptable”* biomedical outcomes. Conversely, when such indicators are perceived as suboptimal or uncertain, relationships may remain more directive or asymmetrical.

This variability suggests that recognition of patient autonomy and expertise is not unconditional, but contingent upon conformity to biomedical expectations. As such, collaboration appears less as a structural feature of care than as a conditional achievement, dependent on the patient’s ability to produce outcomes that align with medical norms.

Taken together, these findings point toward a relational dynamic in which autonomy and recognition are negotiated within a framework that remains strongly influenced by biomedical criteria. This conditional dimension of recognition is further explored in the following theme, particularly in relation to structural and institutional constraints.

4. Persistent Challenges

Despite the emergence of experiential autonomy and, in some cases, more collaborative relationships, participants’ narratives highlight persistent structural and institutional barriers that limit the full integration of their practices within healthcare systems. These challenges reveal that the transformation of the therapeutic relationship remains partial and uneven, shaped by broader constraints beyond individual interactions.

#### Inequalities in access to resources and technologies.

Participants describe significant inequalities in access to technological tools, health information, and the skills required to effectively manage their condition. While some benefit from advanced devices and high levels of health literacy, others face economic, cognitive, or social barriers that restrict their capacity to develop similar forms of autonomy.

As S12 suggests, certain patients appear as *“spoiled children of diabetes”,* while S9 explicitly frames this disparity in terms of social inequality:

*“The dumber you are, the worse you treat yourself… the smarter you are, the more money you have, the better you treat yourself… the healthier you are.”*

These accounts point to a stratification of patient experience, in which autonomy and effective self-management are not equally accessible, but distributed according to broader social determinants. This uneven access challenges the implicit assumption that all patients can equally engage in self-directed care.

At the same time, participants report gaps between technological advancements and professional training. As S13 notes, even healthcare providers may struggle to keep pace with emerging tools:

*“*She was discovering a lot of things too*”,*

and admitted: *“Well, I don’t know”.*

This highlights that disparities are not limited to patients but also affect healthcare systems, contributing to uncertainty and asymmetry in knowledge.

#### Protocol constraints and professional uncertainty.

Participants frequently describe the limits imposed by institutional protocols and the reluctance of healthcare professionals to move beyond established frameworks. In complex or atypical situations, such as combining closed-loop systems with unconventional dietary approaches, clinicians may express uncertainty or hesitation:

*“I’m not sure how we’re going to handle this… there’s no real protocol”* (S9).

This reliance on standardized protocols reflects a tension between the need for safety and the realities of individualized care in extreme contexts. It may limit the capacity of healthcare professionals to support innovative or non-normative practices, even when these are experienced as effective by patients.

More broadly, these findings suggest that institutional structures tend to privilege standardized forms of knowledge and practice, making it difficult to accommodate highly individualized or evolving approaches to care.

#### Unrecognized and informal experiential expertise.

Although the notion of the “expert patient” is formally acknowledged in some therapeutic education programs, most experiential knowledge described by participants is acquired outside institutional settings. This knowledge is often fragmented, informal, and circulated through peer interactions rather than formally integrated into care pathways.

As S4 describes, management strategies are frequently built from “little pieces gathered here and there,” reflecting a cumulative and decentralized process of learning. Similarly, insights gained through peer exchange can significantly reshape understandings of the disease, as illustrated by S6:

*“Hypoglycemia wasn’t about not eating enough—it was about taking too much insulin.”*

These examples suggest that experiential knowledge plays a foundational role in practice yet remains only partially recognized within formal healthcare structures. This gap contributes to a form of epistemic disconnection between what is learned in practice and what is validated in clinical settings.

#### The role of transgression in knowledge construction.

Several participants describe deliberately engaging in practices that were initially discouraged or considered unsafe by healthcare professionals. These *“transgressions”* are not simply acts of resistance but constitute a mode of experimentation through which alternative strategies are tested and validated.

*”I don’t see why someone should tell me what I can or can’t do!” (S13).*

Activities such as canyoning or ski touring, described as *“forbidden at the time”* (S5) were undertaken despite medical advice and subsequently contributed to the development of new forms of knowledge and confidence.

These experiences suggest that stepping outside established norms can play a productive role in expanding the boundaries of what is considered possible or acceptable in diabetes management. However, they also highlight the absence of structured spaces within healthcare systems to safely explore such innovations.

Taken together, these findings indicate that the recognition of experiential knowledge and the development of collaborative care remain constrained by structural inequalities, institutional norms, and epistemic boundaries. These constraints contribute to maintaining asymmetries within the therapeutic relationship, even as new forms of autonomy and collaboration emerge.

## Discussion

Narrative Tensions Between Institutional Medical Knowledge and Experiential Expertise in Type 1 Diabetes and Ultra-Endurance.

This study explored the physician–patient relationship among individuals living with type 1 diabetes engaged in extreme endurance sports, with a focus on the dynamics of autonomy, recognition, and negotiation in care. The findings reveal a persistent tension between institutional medical knowledge and the experiential expertise developed by patients in these contexts.

As illustrated in our results, this tension goes beyond a simple divergence in recommendations. It reflects a deeper misalignment between standardized biomedical frameworks and the situated, experience-based knowledge required in real-world practice. This dynamic challenges the foundations of the therapeutic relationship, the patient’s role in care, and the conditions under which experiential expertise—both personal and peer-recognized—is acknowledged within clinical interactions.

### Medical Expertise Perceived as Incomplete

Our findings indicate that medical expertise is often perceived as incomplete in the specific context of ultra-endurance and type 1 diabetes management. Rather than rejecting medical knowledge altogether, participants describe a mismatch between standardized biomedical frameworks and the situated, experience-based knowledge required to manage prolonged physical exertion.

As illustrated in participants’ accounts of receiving generic or insufficiently tailored advice, this gap reflects broader limitations in medical training when addressing atypical or highly individualized clinical situations. Previous research has similarly highlighted that scientific literature on diabetes and exercise remains largely dominated by biomedical approaches, often disconnected from patients’ lived experiences [16].

In this context, the perceived insufficiency of medical expertise does not lead to disengagement from care, but rather to a redistribution of knowledge production, in which patients develop their own strategies through experimentation and peer exchange. These strategies may at times diverge from official guidelines, not as a form of opposition, but as a pragmatic adaptation to the limits of available medical knowledge.

### Autonomy as Necessity Rather Than Ideological Choice

Our findings suggest that the autonomy described by participants does not stem from an ideological claim to independence but rather emerges as a pragmatic response to the absence of sufficiently tailored medical guidance. As illustrated in participants’ accounts of self-experimentation and adaptation, practices such as personal data tracking, iterative adjustment, and engagement with peer communities become essential for ensuring safe and effective disease management in extreme contexts.

In this sense, autonomy appears less as a normative ideal than as a situated necessity, shaped by the limits of standardized clinical knowledge. These dynamics resonate with scholarship on the “expert patient”, which emphasizes the legitimacy of experiential knowledge, while also highlighting its continued marginalization within formal healthcare structures [17, 18].

These findings are consistent with previous work highlighting the importance of self-monitoring technologies and adaptive strategies in supporting patient-led management in type 1 diabetes [19] while extending this perspective by examining how such experiential expertise is recognized within clinical relationships.

#### Glycemic meritocracy and relational asymmetry: conditional autonomy.

Our findings further indicate that the recognition of this experiential autonomy within clinical relationships remains conditional. As illustrated by participants’ experiences of gaining greater flexibility from healthcare professionals when glycemic outcomes are considered satisfactory, collaboration tends to emerge only once patient competence is demonstrated through biomedical indicators.

This conditional recognition reflects a partial redistribution of medical authority toward a more collaborative model, consistent with contemporary approaches to therapeutic education and person-centered care [20]. However, it also reveals a persistent asymmetry: recognition is not granted uniformly but depends on the patient’s capacity to conform to biomedical expectations.

As suggested by participants’ accounts, only those perceived as “performing” patients—those able to maintain acceptable glycemic indicators—are granted valued autonomy. This introduces an implicit categorization of patients into “good” and “bad” a dynamic well-documented in medical sociology [19].

In this context, we propose the concept of glycemic meritocracy to describe an implicit model in which recognition of patient autonomy, competence, or legitimacy is conditioned by the ability to produce medically acceptable metrics. Biological indicators— such as HbA1c levels, time-in-range, or frequency of hypoglycemia— function not only as clinical tools but also as markers of reliability, shaping clinicians’ trust and attitudes.

Within this framework, the *“*well-balanced*”* patient is perceived as responsible, autonomous, and credible, whereas less favorable indicators may lead to more directive or even moralizing forms of care. This highlights how biomedical norms contribute to structuring not only clinical decisions, but also the distribution of legitimacy and authority within the therapeutic relationship.

### Reinforcing Relational Asymmetry and Normative Care

As illustrated by participants’ experiences of conditional recognition, this dynamic reinforces a form of relational asymmetry in which patient legitimacy is no longer grounded primarily in lived experience, effort, or adaptive strategies, but increasingly in conformity to biomedical norms. In this context, experiential knowledge is only partially recognized and remains subordinated to measurable outcomes.

This configuration echoes the social role of the patient described by Carricaburu and Ménoret, in which implicit expectations within the healthcare system shape the modalities of support [19]. In line with our findings, subjective experience, well-being, and quality of life may be relegated to the background, in favor of a normative reading of care centered on biological performance [21,22].

This reduction of care to performance calls into question the foundations of the therapeutic relationship. It suggests that legitimacy in care is not equally distributed, but conditional upon alignment with expected norms, thereby reinforcing existing asymmetries. At the same time, it highlights tensions identified in our results, where patients must navigate between compliance and adaptation, often negotiating the boundaries of acceptable practice.

These findings invite a broader recognition of singular trajectories—including those marked by trial and error, difficulty, or unconventional choices. They also call for a reconsideration of shared vulnerability: while patients remain exposed to glycemic risks and medical judgment, healthcare professionals face their own forms of uncertainty when confronted with atypical practices and off-protocol situations.

Acknowledging this mutual vulnerability may help shift the therapeutic relationship away from a unidirectional *“*knower–receiver*”* model toward a more dialogical and genuinely collaborative partnership [23].

### Rethinking Autonomy: Between Trust, Responsibility, and Vulnerability

As illustrated by participants’ need to adapt, reinterpret, or sometimes bypass medical recommendations, the findings of this study invite a broader reflection on the limits of the autonomy paradigm as commonly invoked in medicine. In this context, autonomy does not appear as a stable or universally applicable principle, but as a situated process shaped by unequal resources, knowledge, and power.

As Onora O’Neill has argued, an abstract and individualistic conception of autonomy may paradoxically reinforce implicit paternalism by obscuring disparities in capacity, understanding, or power between patients and caregivers [24]. Our findings echo this perspective, showing that autonomy is not simply *“*granted*”* but negotiated, often under conditions that remain structured by biomedical expectations.

O’Neill’s notion of relational autonomy—grounded in trust, shared responsibility, and mutual recognition of vulnerability—resonates with the dynamics observed in this study. Respecting autonomy, in this sense, does not mean withdrawing from the relationship, but rather creating the conditions for dialogue, adjustment, and co-construction in situations characterized by uncertainty [25].

Stirrat and Gill extend this reflection by warning that uncritical adherence to patient requests may constitute a form of bioethical paternalism, which ultimately absolves the caregiver of responsibility [26]. In light of our findings, this highlights the limits of framing the therapeutic relationship as a simple opposition between paternalism and autonomy.

Instead, our results suggest that the physician–patient relationship is better understood as a negotiated space, where responsibility, trust, and knowledge are continuously redistributed. This perspective aligns with the tensions described by participants, who navigate between dependence and autonomy, compliance and adaptation, within a framework that remains shaped by both clinical norms and lived experience.

### Structural Barriers to Recognition

Despite the emergence of more collaborative dynamics, our findings highlight persistent structural and institutional barriers that limit the full integration of experiential knowledge into care. As illustrated by participants’ accounts of uncertainty, unequal access to resources, and difficulties in adapting clinical protocols to atypical situations, these challenges extend beyond individual interactions.

Protocol rigidity, fear of patient harm, unequal access to information and technology, and the absence of formal frameworks for integrating lay expertise all contribute to constraining caregivers’ ability to support so-called *“*pioneer*”* patients. In line with our results, these barriers reflect not only organizational limitations, but also a broader difficulty in moving beyond a biomedical model centered on standardization, safety, and conformity.

The findings further reveal a tension between individual autonomy and collective justice. As As S9 suggests, access to autonomy and recognition is socially stratified: illness becomes *“socially unjust”*, as only patients with sufficient cognitive, financial, or social resources are able to access tools, knowledge, and legitimacy. This dynamic reinforces the logic of glycemic meritocracy identified in our study, whereby “performing” patients—those able to produce favorable biomedical indicators—are more likely to be recognized as competent and trustworthy, while others remain marginalized. In this sense, autonomy appears not as a universal principle, but as a differential resource, unevenly distributed across patient populations.

Such a configuration resembles a form of selective paternalism, in which autonomy is granted conditionally, based on perceived competence [27]. As illustrated in our findings, this raises important ethical questions: to what extent can autonomy legitimately depend on adherence to caregiver-defined norms? And to what extent might subjective well-being or individual trajectories be overlooked in favor of biological performance?

Several authors have called for alternative approaches to these challenges. Weidmann-Hügle and Monteverde propose the notion of epistemic modesty, summarized by the stance “I know nothing”, which implies a redistribution of knowledge toward the patient [28]. Similarly, Sullivan suggests that the concept of capability may offer a more inclusive and context-sensitive understanding of health in chronic illness [29].

### Limitations and Potential Biases

This study has several limitations that should be acknowledged. First, the sample is composed exclusively of French-speaking runners living with type 1 diabetes, which may limit the transferability of findings to other linguistic, cultural, or healthcare contexts. Second, the principal investigator may have influenced both data collection and interpretation. While this insider position facilitated access, trust, and depth of insight, it also required sustained reflexivity to mitigate potential bias, as described in the Methods section. Third, participants were recruited through peer networks and social media, which may have favored individuals already engaged in self-management and community exchange, potentially underrepresenting more isolated or vulnerable patients.

Finally, the concept of glycemic meritocracy, while grounded in empirical data, remains interpretive and would benefit from further exploration and validation in broader populations and clinical contexts.

### Implications for Primary Care and Broader Perspectives on Autonomy

While this study focused on the context of type 1 diabetes and ultra-endurance sports, its findings have broader relevance for primary care. General practitioners are often the first point of contact for patients managing complex chronic conditions, and they play a pivotal role in supporting patient autonomy, recognizing lay expertise, and facilitating collaborative care. The dynamics of conditional autonomy and the challenges of integrating experiential knowledge, as described here, are not unique to diabetes. Similar tensions may arise in the management of other chronic diseases—such as asthma, rheumatoid arthritis, or cardiovascular conditions—where patients develop individualized strategies that may diverge from standard protocols. This raises important questions for primary care: Is autonomy always ‘earned’ through biomedical performance, or should it be recognized as a fundamental right across all chronic pathologies? Future research could explore how the concept of ‘meritocratic’ autonomy manifests in other clinical contexts, and how primary care teams can foster more inclusive, patient-centered approaches that value diverse forms of expertise and experience.

## Conclusion

At the heart of these dynamics lies an implicit logic we propose to call glycemic meritocracy: a model in which patient recognition, trust, and autonomy are conditioned by conformity to glycemic norms. This concept offers a novel lens for examining power relations in care, showing how legitimacy is not equally distributed but depends on patients’ ability to produce medically acceptable outcomes.

These findings have important clinical implications. They suggest that care practices should move beyond a strictly biomedical evaluation of patients and more explicitly integrate experiential knowledge, particularly in complex or atypical situations. This includes acknowledging uncertainty, supporting shared decision-making, and valuing diverse trajectories of disease management rather than privileging standardized performance indicators alone.

More broadly, this study invites a rethinking of autonomy, not as an individual attribute to be granted or withheld, but as a relational and situated process shaped by interactions, resources, and institutional norms. The ultra-endurance context thus provides a revealing lens for understanding broader tensions in chronic illness care.

Ultimately, fostering genuine health democracy requires moving beyond the mere tolerance of experiential knowledge toward its full integration into care, training, and research systems.

This qualitative study explored the physician–patient relationship among individuals living with type 1 diabetes engaged in extreme endurance sports, focusing on the dynamics of autonomy, recognition, and negotiation in care.

The findings highlight persistent tensions between institutional medical knowledge and experiential expertise, structured by gaps in expertise, the emergence of experiential autonomy, conditional recognition, and enduring structural constraints.

At the heart of this dynamic lies an implicit logic we propose to call glycemic meritocracy: a model in which patient recognition, trust, and autonomy are conditioned by conformity to glycemic norms. This concept offers a novel lens for examining power relations in care, showing how legitimacy is not equally distributed but depends on patients’ ability to produce medically acceptable outcomes.

These findings have important clinical implications. They suggest that care practices should move beyond a strictly biomedical evaluation of patients and more explicitly integrate experiential knowledge, particularly in complex or atypical situations. This includes acknowledging uncertainty, supporting shared decision-making, and valuing diverse trajectories of disease management rather than privileging standardized performance indicators alone.

More broadly, this study invites a rethinking of autonomy, not as an individual attribute to be granted or withheld, but as a relational and situated process shaped by interactions, resources, and institutional norms. The ultra-endurance context thus provides a revealing lens for understanding broader tensions in chronic illness care.

Ultimately, fostering genuine health democracy requires moving beyond the mere tolerance of experiential knowledge toward its full integration into care, training, and research systems.

## Supporting information

S1 File COREQ_Checklist. (DOCX)
