# Supplementary material for: Glycemic meritocracy, sport and care: Between lay expertise and medical recognition
Source: PLoS One. 2026 Jul 14;21(7):e0353885. doi: 10.1371/journal.pone.0353885 (PMC13367717; doi:10.1371/journal.pone.0353885)
Supplement: S1 File — (DOCX) [file pone.0353885.s001.docx]

# COREQ Checklist (Consolidated Criteria for Reporting Qualitative Research)

## Domain 1: Research team and reflexivity

- 1. Interviewer/facilitator: Yes, specified (principal investigator).
- 2. Credentials: General practitioner, university lecturer-researcher, thesis supervisor in medicine.
- 3. Occupation: Academic general practitioner at the time of the study.
- 4. Gender: Male.
- 5. Experience and training: Experienced in qualitative research, trained in Grounded Theory and reflexivity.
- 6. Relationship established: Yes, prior immersion in peer support groups.
- 7. Participant knowledge of the interviewer: Yes, identity and background disclosed.
- 8. Interviewer characteristics: Reflexive posture, personal experience with type 1 diabetes and ultra-endurance sport.

## Domain 2: Study design

- 9. Methodological orientation and theory: Constructivist Grounded Theory (Charmaz).
- 10. Sampling: Theoretical sampling, adjusted iteratively.
- 11. Method of approach: Social media, word-of-mouth, peer networks.
- 12. Sample size: 13 participants.
- 13. Non-participation: None reported.
- 14. Setting of data collection: Videoconference.
- 15. Presence of non-participants: No.
- 16. Description of sample: Adults with type 1 diabetes, French-speaking, engaged in endurance sports.
- 17. Interview guide: Yes, enriched progressively.
- 18. Repeat interviews: No.
- 19. Audio/visual recording: Audio only, destroyed after transcription.
- 20. Field notes: Yes, research journal maintained.
- 21. Duration: Average of 57 minutes.
- 22. Data saturation: Pragmatic approach, reasoned stopping point.
- 23. Transcripts returned: No.

## Domain 3: Analysis and findings

- 24. Number of data coders: Collaborative analysis with triangulation.
- 25. Description of the coding tree: Inductive coding, axial coding, categorization, theorization.
- 26. Derivation of themes: Yes, thematic structuring.
- 27. Software: NVivo®.
- 28. Participant checking: No.
- 29. Quotations presented: Yes, anonymized excerpts.
- 30. Data and findings consistent: Strong consistency, well-argued.
- 31. Clarity of major themes: Yes.
- 32. Clarity of minor themes: Yes, diversity acknowledged despite sociological homogeneity.
