# Supplementary material for: Glycemic meritocracy, sport and care: Between lay expertise and medical recognition
Source: PLoS One. 2026 Jul 14;21(7):e0353885. doi: 10.1371/journal.pone.0353885 (PMC13367717; doi:10.1371/journal.pone.0353885)
Supplement: Attachment — Submitted filename: Response to Reviewers.docx [file pone.0353885.s003.docx]

Response to Reviewers

Dear Academic Editor and Reviewers,

We thank you for your careful evaluation of our manuscript and for your constructive and insightful comments. We greatly appreciate the time and expertise you have devoted to improving our work.

In response to your feedback, we have thoroughly revised the manuscript. We have clarified the research aim and theoretical framing in the Introduction, substantially expanded and refined the Methods section to improve transparency and rigor, and strengthened the analytical depth and coherence of the Results and Discussion sections. In particular, we have developed a more explicit articulation of the concept of “glycemic meritocracy” and its empirical grounding.

All revisions have been made with tracked changes in the manuscript. Below, we provide a point-by-point response to each comment.

We hope that these revisions satisfactorily address your concerns and improve the clarity and quality of the manuscript.

Sincerely,

Jean-Charles Vauthier

On behalf of all authors

**Responses to reviewer 1**

Comment: The introduction does not clearly articulate the research problem, gap, and study aim.

Response: We thank the reviewer for this important comment. We have revised the Introduction to more explicitly articulate the research problem, the gap in the literature, and the aim of the study. In particular, we now clearly state the lack of attention to the relational and experiential dimensions of the physician–patient relationship in extreme contexts, and we explicitly present the study aim and research question at the end of the Introduction (see revised manuscript, Introduction section).

Comment: The methods section lacks sufficient transparency.

Response: We agree with the reviewer and have substantially revised and expanded the Methods section to improve clarity and transparency. Specifically, we have:

- Clarified our use of constructivist grounded theory and its epistemological foundations

- Explicitly described the sampling strategy (purposive sampling with maximum variation)

- Detailed the data collection procedures and interview content

- Expanded the description of the analytical process, including coding steps, memo-writing, and team discussions

- Clarified the notion of interpretive sufficiency (saturation)

- Strengthened the reflexivity section to better account for the researcher’s insider position

These revisions aim to enhance methodological rigor and align the manuscript with qualitative research reporting standards.

Comment: The results remain largely descriptive and lack analytical depth.

Response: We thank the reviewer for this insightful comment. We have revised the Results section to strengthen its analytical depth and conceptual coherence. Specifically, we have:

- Reduced redundancy with the Methods section

- Added interpretative elements throughout the thematic analysis

- Strengthened the conceptual framing of each theme

- Improved the integration between themes by highlighting their interrelations

- Added synthesis statements at the end of each section

These revisions aim to move beyond description toward a more interpretive and theoretically informed presentation of the findings.

Comment: The discussion occasionally overextends beyond the data and lacks consistent grounding.

Response: We appreciate this comment and have revised the Discussion accordingly. We have strengthened the link between empirical findings and theoretical interpretation by systematically anchoring key arguments in the data (e.g., adding expressions such as “as illustrated in our findings”). We have also slightly reduced the density of theoretical exposition in order to improve balance between data and interpretation.

Comment: The conclusion does not fully articulate the study’s contribution.

Response: We have revised the Conclusion to more clearly restate the research aim, synthesize the main findings, and explicitly highlight the contribution of the concept of “glycemic meritocracy.” We have also strengthened the practical implications to provide a clearer response to the “so what” of the study.

**Responses to Reviewer 2**

Comment: The theoretical framework seems inconsistent with grounded theory.

Response: We thank the reviewer for this important observation. We have clarified our methodological positioning by explicitly stating our use of constructivist grounded theory (Charmaz), which allows for the integration of existing theoretical perspectives while maintaining an inductive and iterative analytical approach. We have revised the Introduction to better align the theoretical framework with this methodological stance and clarified this point in the Methods section.

Comment: Provide more details on sampling, analysis, and rigor.

Response: We fully agree and have substantially expanded the Methods section to address these points. In particular, we have clarified:

- The type of study and theoretical orientation

- The sampling strategy and inclusion criteria

- The data collection process and interview guide

- The analytical procedure (coding steps and category development)

- The rationale for sample size and interpretive sufficiency

- The strategies used to ensure rigor (reflexivity, triangulation)

These additions aim to improve transparency and reproducibility.

Comment: The findings lack diversity of voices.

Response: We thank the reviewer for this suggestion. We have revised the Results section to better reflect variations across participants, including the introduction of contrasting experiences and nuances within themes. This includes highlighting differences in levels of autonomy, dependence on medical guidance, and access to resources.

Comment: Replace discussion opening with study aim.

Response: We have revised the beginning of the Discussion to explicitly restate the study aim before presenting the main interpretative argument.

**Additional editorial requirements:**

We have addressed all editorial comments, including formatting, title page, ethics statement placement, table inclusion (Table 1), and revision of the data availability statement as requested.
