# Supplementary material for: Glycemic meritocracy, sport and care: Between lay expertise and medical recognition
Source: PLoS One. 2026 Jul 14;21(7):e0353885. doi: 10.1371/journal.pone.0353885 (PMC13367717; doi:10.1371/journal.pone.0353885)
Supplement: Peer review history

# Peer review: Decision Letter 0

**DOI:** 10.1371/journal.pone.0353885.r001

**Authors:** Hidetaka Hamasaki

13 Jun 2026

PONE-D-25-55203

Glycemic Meritocracy, Sport and Care: Between Lay Expertise and Medical Recognition

PLOS One

Dear Dr. VAUTHIER,

Thank you for submitting your manuscript to PLOS ONE. After careful consideration, we feel that it has merit but does not fully meet PLOS ONE’s publication criteria as it currently stands. Therefore, we invite you to submit a revised version of the manuscript that addresses the points raised during the review process.

Please submit your revised manuscript by Jul 28 2026 11:59PM. If you will need more time than this to complete your revisions, please reply to this message or contact the journal office at plosone@plos.org. When you're ready to submit your revision, log on to https://www.editorialmanager.com/pone/ and select the 'Submissions Needing Revision' folder to locate your manuscript file.

Please include the following items when submitting your revised manuscript:

- A letter that responds to each point raised by the academic editor and reviewer(s). You should upload this letter as a separate file labeled 'Response to Reviewers'.
- A marked-up copy of your manuscript that highlights changes made to the original version. You should upload this as a separate file labeled 'Revised Manuscript with Track Changes'.
- An unmarked version of your revised paper without tracked changes. You should upload this as a separate file labeled 'Manuscript'.

If you would like to make changes to your financial disclosure, please include your updated statement in your cover letter. Guidelines for resubmitting your figure files are available below the reviewer comments at the end of this letter.

If applicable, we recommend that you deposit your laboratory protocols in protocols.io to enhance the reproducibility of your results. Protocols.io assigns your protocol its own identifier (DOI) so that it can be cited independently in the future. For instructions see: https://journals.plos.org/plosone/s/submission-guidelines#loc-laboratory-protocols. Additionally, PLOS ONE offers an option for publishing peer-reviewed Lab Protocol articles, which describe protocols hosted on protocols.io. Read more information on sharing protocols at https://plos.org/protocols?utm_medium=editorial-email&utm_source=authorletters&utm_campaign=protocols.

As the corresponding author, your ORCID iD is verified in the submission system and will appear in the published article. PLOS supports the use of ORCID, and we encourage all coauthors to register for an ORCID iD and use it as well. Please encourage your coauthors to verify their ORCID iD within the submission system before final acceptance, as unverified ORCID iDs will not appear in the published article. *Only*  the individual author can complete the verification step; PLOS staff *cannot*  verify ORCID iDs on behalf of authors.

We look forward to receiving your revised manuscript.

Kind regards,

Hidetaka Hamasaki

Academic Editor

PLOS One

**Journal Requirements:**

When submitting your revision, we need you to address these additional requirements.

1. Please ensure that your manuscript meets PLOS ONE's style requirements, including those for file naming. The PLOS ONE style templates can be found at

https://journals.plos.org/plosone/s/file?id=wjVg/PLOSOne_formatting_sample_main_body.pdf and

https://journals.plos.org/plosone/s/file?id=ba62/PLOSOne_formatting_sample_title_authors_affiliations.pdf

2. In the online submission form you indicate that your data is not available for proprietary reasons and have provided a contact point for accessing this data. Please note that your current contact point is a co-author on this manuscript. According to our Data Policy, the contact point must not be an author on the manuscript and must be an institutional contact, ideally not an individual. Please revise your data statement to a non-author institutional point of contact, such as a data access or ethics committee, and send this to us via return email. Please also include contact information for the third party organization, and please include the full citation of where the data can be found.

3. Please ensure that you include a title page within your main document. You should list all authors and all affiliations as per our author instructions and clearly indicate the corresponding author.

4. Your ethics statement should only appear in the Methods section of your manuscript. If your ethics statement is written in any section besides the Methods, please delete it from any other section.

5. Please include a copy of Table 1 which you refer to in your text on page 9.

6. If the reviewer comments include a recommendation to cite specific previously published works, please review and evaluate these publications to determine whether they are relevant and should be cited. There is no requirement to cite these works unless the editor has indicated otherwise.

[Note: HTML markup is below. Please do not edit.]

Reviewers' comments:

Reviewer's Responses to Questions

**Comments to the Author**

1. Is the manuscript technically sound, and do the data support the conclusions?

The manuscript must describe a technically sound piece of scientific research with data that supports the conclusions. Experiments must have been conducted rigorously, with appropriate controls, replication, and sample sizes. The conclusions must be drawn appropriately based on the data presented.

Reviewer #1: No

Reviewer #2: Partly

**********

2. Has the statistical analysis been performed appropriately and rigorously?

Reviewer #1: N/A

Reviewer #2: N/A

**********

3. Have the authors made all data underlying the findings in their manuscript fully available?

The PLOS Data policy requires authors to make all data underlying the findings described in their manuscript fully available without restriction, with rare exception (please refer to the Data Availability Statement in the manuscript PDF file). The data should be provided as part of the manuscript or its supporting information, or deposited to a public repository. For example, in addition to summary statistics, the data points behind means, medians and variance measures should be available. If there are restrictions on publicly sharing data—e.g. participant privacy or use of data from a third party—those must be specified.

Reviewer #1: Yes

Reviewer #2: No

**********

4. Is the manuscript presented in an intelligible fashion and written in standard English?

PLOS ONE does not copyedit accepted manuscripts, so the language in submitted articles must be clear, correct, and unambiguous. Any typographical or grammatical errors should be corrected at revision, so please note any specific errors here.

Reviewer #1: Yes

Reviewer #2: No

**********

5. Review Comments to the Author

Please use the space provided to explain your answers to the questions above. You may also include additional comments for the author, including concerns about dual publication, research ethics, or publication ethics. (Please upload your review as an attachment if it exceeds 20,000 characters)

Reviewer #1: This manuscript addresses an important and underexplored area at the intersection of chronic illness management and extreme endurance sport, and it offers a thoughtful and conceptually rich analysis of the therapeutic relationship in this context. The empirical material is compelling, and the introduction of the concept of “glycemic meritocracy” is a promising and original contribution that has the potential to advance discussions around autonomy, expertise, and power in healthcare. The manuscript is generally well structured, and the discussion demonstrates strong engagement with relevant theoretical and ethical literature. However, the paper would benefit from substantial revisions before it is ready for publication. In particular, the introduction does not clearly articulate the research problem, gap, and study aim, which weakens the overall framing. The methods section lacks sufficient transparency, especially regarding sampling strategy, reflexivity, and analytic procedures. The results, while rich, remain largely descriptive and would benefit from stronger conceptual synthesis. The discussion, although insightful, occasionally overextends beyond the data and would be strengthened by tighter integration between empirical findings and theoretical claims. Finally, the conclusion and implications remain somewhat general and do not fully convey the significance and practical relevance of the study. Overall, this is a strong and promising manuscript, but it requires major revision to sharpen its analytical clarity, methodological rigour, and contribution to the field. For better clarify below are the review points:

Introduction:

Research focus unclear: The central focus is implied but not explicitly stated.

Research problem implicit: Tensions between biomedical and experiential knowledge are identified but not clearly formulated as a problem.

Research gap under-specified: The lack of focus on relational, subjective, and identity dimensions is noted but not sharply articulated.

Theoretical link needs clarity: Use of Carl Rogers, Paul Ricoeur, Joan Tronto, and Emmanuel Levinas is strong but not clearly connected to the empirical case.

Rationale diffuse: The need and contribution of the study are not explicitly stated.

Missing study aim: No clear aim or research questions provided.

Overall: Conceptually strong, but requires sharper articulation of problem, gap, and aim.

Methods:

Design: Appropriate, but how co-construction shaped analysis is not shown.

Positioning: Insider role stated, but no clear reflexive impact on findings.

Sampling: Recruitment described, but sampling strategy not named (purposive/theoretical)

Sample: Gender imbalance acknowledged nowhere.

Data collection: No interview duration, guide, or core themes provided.

Non-verbal data: Mentioned, but method of capture unclear.

Analysis: Steps listed, but no example of coding - category development.

Triangulation: Stated, but who did what is unclear.

Saturation: Claimed, but no criteria or evidence given.

NVivo: Mentioned, but adds no analytical value explanation.

Ethics: Clear and sufficient.

Overall: Method is appropriate but lacks transparency and analytical depth.

Results:

Descriptive overview clear: Sample and interview details are well presented.

Analysis section repetitive: Repeats methods (coding, NVivo, saturation) instead of focusing on findings.

Themes clearly identified: Four themes are coherent, but not conceptually framed.

Theme structure inconsistent: Some themes are analytical (e.g., autonomy), others are descriptive (e.g., challenges).

Over-reliance on quotes: Rich data, but quotes dominate over interpretation.

Limited analytical depth: Findings remain descriptive, not theoretically engaged.

Weak integration across themes: Themes are presented separately with no synthesis or relational linking.

Conceptual contribution unclear: No clear articulation of what new understanding emerges.

Power dynamics implicit: Doctor-patient hierarchy appears, but not explicitly analysed.

Experiential knowledge strong but under-theorised: Not clearly framed as epistemic or relational shift.

Gender imbalance ignored: No reflection on 11 men vs 2 women in findings.

Transition to discussion missing: Results do not prepare analytical argument for next section.

Overall

Empirically rich and well-organised, but needs stronger analytical interpretation, thematic coherence, and conceptual synthesis to meet high-level qualitative research standards.

Discussion:

Central argument strong and consistent: Clearly sustains the tension between institutional and experiential knowledge .

Conceptual contribution improved: “Glycemic meritocracy” is now clearer, but still needs sharper theoretical anchoring within existing frameworks.

Analytical progression better: Moves more logically across sections, though some repetition remains (autonomy/asymmetry).

Data–theory linkage improved but uneven: Key claims are better grounded, yet some abstract arguments still lack direct empirical anchoring.

Theoretical integration still dense: Strong engagement, but occasionally dominates over data-driven insight.

Conceptual clarity stronger: Terms like autonomy and asymmetry are clearer, but boundaries between them still blur at points.

“So what” slightly improved: Ethical and conceptual implications clearer, but practical implications remain general and not actionable.

Power and hierarchy better articulated: “Relational asymmetry” more explicit, but could be further deepened analytically.

Structural flow improved: Clearer sections, though minor redundancy persists.

Limitations and reflexivity well handled: Strong and transparent.

Implications relevant but broad: Primary care section useful, but needs more concrete recommendations.

Conclusion:

The conclusion is conceptually strong and coherent, effectively synthesising key findings and foregrounding the original contribution of “glycemic meritocracy” while extending the discussion toward broader ethical and clinical implications. It maintains alignment with the overall argument and offers a confident closing. However, it does not explicitly restate or resolve the research problem, leaving the analytical loop partially open. The research gap is not clearly closed, and the central concept, while present, is not fully consolidated as the main theoretical contribution. Additionally, the implications remain broad and lack concrete, actionable direction, which weakens the “so what” impact. Overall, it is a solid conclusion but would benefit from sharper articulation of contribution and more precise implications.

Reviewer #2: Dear Author

Congratulations on being a good voice for athletes with type 1 diabetes. I noticed that the main author also has the same disease and is an endurance athlete and has a common experience with the participants. I wish you health and success.

Sheka's findings can add to the body of knowledge in the management of type 1 diabetes in these individuals. Of course, I have suggested the following items to improve the manuscript, which I hope will be considered by you.

Abstract

-Method: I suggest that the method of selecting participants be specified.

-Findings: Present the findings with more details, including explanation of categories and subcategories.

Conclusion: Review based on the findings of the study. Conclusions are presented beyond the findings of this study.

Introduction:

- The beginning of the introduction is well written and recounts the communication problems between health workers and endurance athletes with type 1 diabetes. But in the end, the necessity of conducting a grounded theory study is not clear. There is a predetermined theoretical framework that is not in line with the principles of grounded theory. You can use the explained theoretical framework in the conclusion.

The purpose and research question should be presented clearly at the end of the problem statement.

Method:

This section of the paper is not written well and clearly.

I suggest that you provide more details; the type of study (the researcher's philosophical orientation and justification for choosing structuralist grounded theory), the method of selecting study participants, the reasons for selecting 13 participants in the study, the study area, the method or methods of data collection, the method of data analysis (by providing a flowchart of the analysis process), the method of ensuring the robustness and rigor of the study.

Also, I have this question: why is the entry criterion of 18 years considered but people over 26 years of age participate in the study? Please explain the reasons and mention it in the limitations.

Findings:

I suggest revising the first paragraph where you presented demographic findings. Delete the sentence “Interviews lasted an average of 57 minutes (minimum: 47, maximum: 68).” and move it to the method. Also, move the explanations provided in lines 11-115 to the method and remove them from the findings.

The categories are presented superficially and lack contextual detail. I suggest that you explain the categories in more detail in the context of the dynamic of the care interaction between the athlete with type 1 diabetes and the health care staff.

I did not hear different voices in the categories constructed based on the experiences of the athlete with type 1 diabetes in the communication between themselves and the health care staff. I suggest that the contradictory voices of the participants be recounted, which represent the dimensions and characteristics of the categories and also provide a guarantee for interpretive validity.

Discussion

I suggest removing "Narrative Tensions Between Institutional Medical Knowledge and Experiential Expertise in Type 1

257 Diabetes and Ultra-Endurance" from the beginning of the discussion and replacing it with the purpose of the study.

Provide your suggestions for future studies

**********

6. PLOS authors have the option to publish the peer review history of their article (what does this mean?). If published, this will include your full peer review and any attached files.

If you choose “no”, your identity will remain anonymous but your review may still be made public.

**Do you want your identity to be public for this peer review?**  For information about this choice, including consent withdrawal, please see our Privacy Policy.

Reviewer #1: No

Reviewer #2: **Yes:** Mehrsadat Mahdizadeh

**********

[NOTE: If reviewer comments were submitted as an attachment file, they will be attached to this email and accessible via the submission site. Please log into your account, locate the manuscript record, and check for the action link "View Attachments". If this link does not appear, there are no attachment files.]

To ensure your figures meet our technical requirements, please review our figure guidelines: https://journals.plos.org/plosone/s/figures

You may also use PLOS’s free figure tool, NAAS, to help you prepare publication quality figures: https://journals.plos.org/plosone/s/figures#loc-tools-for-figure-preparation.

NAAS will assess whether your figures meet our technical requirements by comparing each figure against our figure specifications.

# Author comment: Author response to Decision Letter 1

**DOI:** 10.1371/journal.pone.0353885.r002

16 Jun 2026

We thank the Academic Editor and the reviewers for their thoughtful and constructive comments.We have carefully revised the manuscript to address all concerns raised. Major revisions include:- Clarification of the research aim, problem, and gap in the Introduction - Substantial expansion and clarification of the Methods section, including sampling, reflexivity, and analytical procedures - Strengthening of the analytical depth and coherence of the Results section - Improved integration between empirical findings and theoretical interpretation in the Discussion - Clearer articulation of the concept of “glycemic meritocracy” and its contribution - Refinement of the Conclusion and practical implications A detailed point-by-point response to each comment is provided in the separate “Response to Reviewers” document.

Attachment Submitted filename: Response to Reviewers.docx

# Peer review: Decision Letter 1

**DOI:** 10.1371/journal.pone.0353885.r003

**Authors:** Hidetaka Hamasaki, Hidetaka Hamasaki

30 Jun 2026

Glycemic Meritocracy, Sport and Care: Between Lay Expertise and Medical Recognition

PONE-D-25-55203R1

Dear Dr. VAUTHIER,

We’re pleased to inform you that your manuscript has been judged scientifically suitable for publication and will be formally accepted for publication once it meets all outstanding technical requirements.

Within one week, you’ll receive an e-mail detailing the required amendments. When these have been addressed, you’ll receive a formal acceptance letter and your manuscript will be scheduled for publication.

An invoice will be generated when your article is formally accepted. Please note, if your institution has a publishing partnership with PLOS and your article meets the relevant criteria, all or part of your publication costs will be covered. Please make sure your user information is up-to-date by logging into Editorial Manager at Editorial Manager® and clicking the ‘Update My Information' link at the top of the page. For questions related to billing, please contact billing support.

If your institution or institutions have a press office, please notify them about your upcoming paper to help maximize its impact. If they’ll be preparing press materials, please inform our press team as soon as possible -- no later than 48 hours after receiving the formal acceptance. Your manuscript will remain under strict press embargo until 2 pm Eastern Time on the date of publication. For more information, please contact onepress@plos.org.

Kind regards,

Hidetaka Hamasaki

Academic Editor

PLOS One

Additional Editor Comments (optional):

Thank you for submitting the revised manuscript.

On behalf of Reviewer 1, I reviewed the responses to the reviewers’ comments and the revised sections. Overall, I believe that the authors have addressed the comments adequately.

Reviewers' comments:

Reviewer's Responses to Questions

**Comments to the Author**

1. If the authors have adequately addressed your comments raised in a previous round of review and you feel that this manuscript is now acceptable for publication, you may indicate that here to bypass the “Comments to the Author” section, enter your conflict of interest statement in the “Confidential to Editor” section, and submit your "Accept" recommendation.

Reviewer #2: All comments have been addressed

**********

2. Is the manuscript technically sound, and do the data support the conclusions?

The manuscript must describe a technically sound piece of scientific research with data that supports the conclusions. Experiments must have been conducted rigorously, with appropriate controls, replication, and sample sizes. The conclusions must be drawn appropriately based on the data presented.

Reviewer #2: Yes

**********

3. Has the statistical analysis been performed appropriately and rigorously?

Reviewer #2: N/A

**********

4. Have the authors made all data underlying the findings in their manuscript fully available?

The PLOS Data policy requires authors to make all data underlying the findings described in their manuscript fully available without restriction, with rare exception (please refer to the Data Availability Statement in the manuscript PDF file). The data should be provided as part of the manuscript or its supporting information, or deposited to a public repository. For example, in addition to summary statistics, the data points behind means, medians and variance measures should be available. If there are restrictions on publicly sharing data—e.g. participant privacy or use of data from a third party—those must be specified.

Reviewer #2: Yes

**********

5. Is the manuscript presented in an intelligible fashion and written in standard English?

PLOS ONE does not copyedit accepted manuscripts, so the language in submitted articles must be clear, correct, and unambiguous. Any typographical or grammatical errors should be corrected at revision, so please note any specific errors here.

Reviewer #2: Yes

**********

6. Review Comments to the Author

Please use the space provided to explain your answers to the questions above. You may also include additional comments for the author, including concerns about dual publication, research ethics, or publication ethics. (Please upload your review as an attachment if it exceeds 20,000 characters)

Reviewer #2: Dear Authors,

Thank you for your efforts to improve your manuscript, which contains valuable findings to advance the body of knowledge in the management of type 1 diabetes in athletes.

**********

7. PLOS authors have the option to publish the peer review history of their article (what does this mean?). If published, this will include your full peer review and any attached files.

If you choose “no”, your identity will remain anonymous but your review may still be made public.

**Do you want your identity to be public for this peer review?**  For information about this choice, including consent withdrawal, please see our Privacy Policy.

Reviewer #2: **Yes:** Mehrsadat Mahdizadeh

**********

# Editor report: Acceptance letter

**DOI:** 10.1371/journal.pone.0353885.r004

**Authors:** Hidetaka Hamasaki, Hidetaka Hamasaki

PONE-D-25-55203R1

PLOS One

Dear Dr. VAUTHIER,

I'm pleased to inform you that your manuscript has been deemed suitable for publication in PLOS One. Congratulations! Your manuscript is now being handed over to our production team.

At this stage, our production department will prepare your paper for publication. This includes ensuring the following:

* All references, tables, and figures are properly cited

* All relevant supporting information is included in the manuscript submission,

* There are no issues that prevent the paper from being properly typeset

You will receive further instructions from the production team, including instructions on how to review your proof when it is ready. Please keep in mind that we are working through a large volume of accepted articles, so please give us a few days to review your paper and let you know the next and final steps.

Lastly, if your institution or institutions have a press office, please let them know about your upcoming paper now to help maximize its impact. If they'll be preparing press materials, please inform our press team within the next 48 hours. Your manuscript will remain under strict press embargo until 2 pm Eastern Time on the date of publication. For more information, please contact onepress@plos.org.

You will receive an invoice from PLOS for your publication fee after your manuscript has reached the completed accept phase. If you receive an email requesting payment before acceptance or for any other service, this may be a phishing scheme. Learn how to identify phishing emails and protect your accounts at https://explore.plos.org/phishing.

If we can help with anything else, please email us at customercare@plos.org.

Thank you for submitting your work to PLOS ONE and supporting open access.

Kind regards,

PLOS ONE Editorial Office Staff

on behalf of

Dr. Hidetaka Hamasaki

Academic Editor

PLOS One
